# Hearing objects move: apparent motion in complex sounds

**DOI:** 10.3389/fpsyg.2026.1833706

**Published:** 2026-05-28

**Authors:** Meike C. Kriegeskorte, Bettina Rolke, Elisabeth Hein

**Affiliations:** Department of Psychology, University of Tübingen, Tübingen, Germany

**Keywords:** apparent motion, auditory perception, grouping, motion correspondence, perceptual organization, Ternus display

## Abstract

How can we perceive objects as moving based on auditory information? This information is often not continuous, such as when objects are temporarily masked by other, louder objects. To perceive these auditory objects as moving along a continuous path, rather than as one object disappearing and another one reappearing, we need to establish correspondence between the correct object instances, resulting in an auditory apparent motion percept of only one object moving across space. However, correctly linking the object instances that belong to the same object is challenging, as auditory input is often ambiguous, such as when several objects follow overlapping trajectories. Previous studies with visual objects have shown that correspondence is influenced by spatiotemporal factors and object features, such as the color, size or shape. Recent studies have shown that correspondence in the auditory modality also depends on spatiotemporal and even feature information, i.e., the frequency of simple sinewave tones. This study aimed to determine whether this finding generalizes to more complex sounds that are meaningful to us and which may involve higher levels of processing. To test this idea, we created an ambiguous apparent motion display biased with sounds from two different musical instruments, a piano and a guitar. Additionally, we manipulated spatiotemporal information. Our results demonstrate that the auditory feature bias, which was previously observed with sinewaves, extends to complex sounds. Furthermore, spatiotemporal factors had a weaker influence on correspondence for complex than for simple sounds. This suggests that auditory correspondence might involve different types of correspondence mechanisms.

## 1 Introduction

The world is rarely static: Objects shift, flicker, and vanish. Instead of perceiving a wild mess of different object instances, we recognize ordered objects that move across space. This is only possible, because our sensory systems can connect different instances of one and the same object based on object-specific information, such as the visual appearance, tactile feedback and auditory cues. In doing so, if an object appears at different times in different locations, the sensory systems integrate object-specific information across space and time, establishing correspondence between the different object instances (e.g., Ullman, 1979). As a result, we perceive the object as moving from one location to another. This works even when our perception of the objects is not continuous, as for example our gaze changes or an object is temporarily occluded by other objects. For example, we see many different cars on the road and people on the pavement, and we are usually quite sure about which object has moved and where it has moved to. To connect different instances of objects that belong together the sensory systems could rely on the direction in which the cars are travelling or the people are walking, the perceived speed, or even the shape or sound of the objects. This ability to perceive and interpret sensory inputs and their motions is crucial for understanding and interacting with the environment surrounding us. For this reason, it is important to understand how our sensory systems accurately establish correspondences between objects.

**Figure 1:**
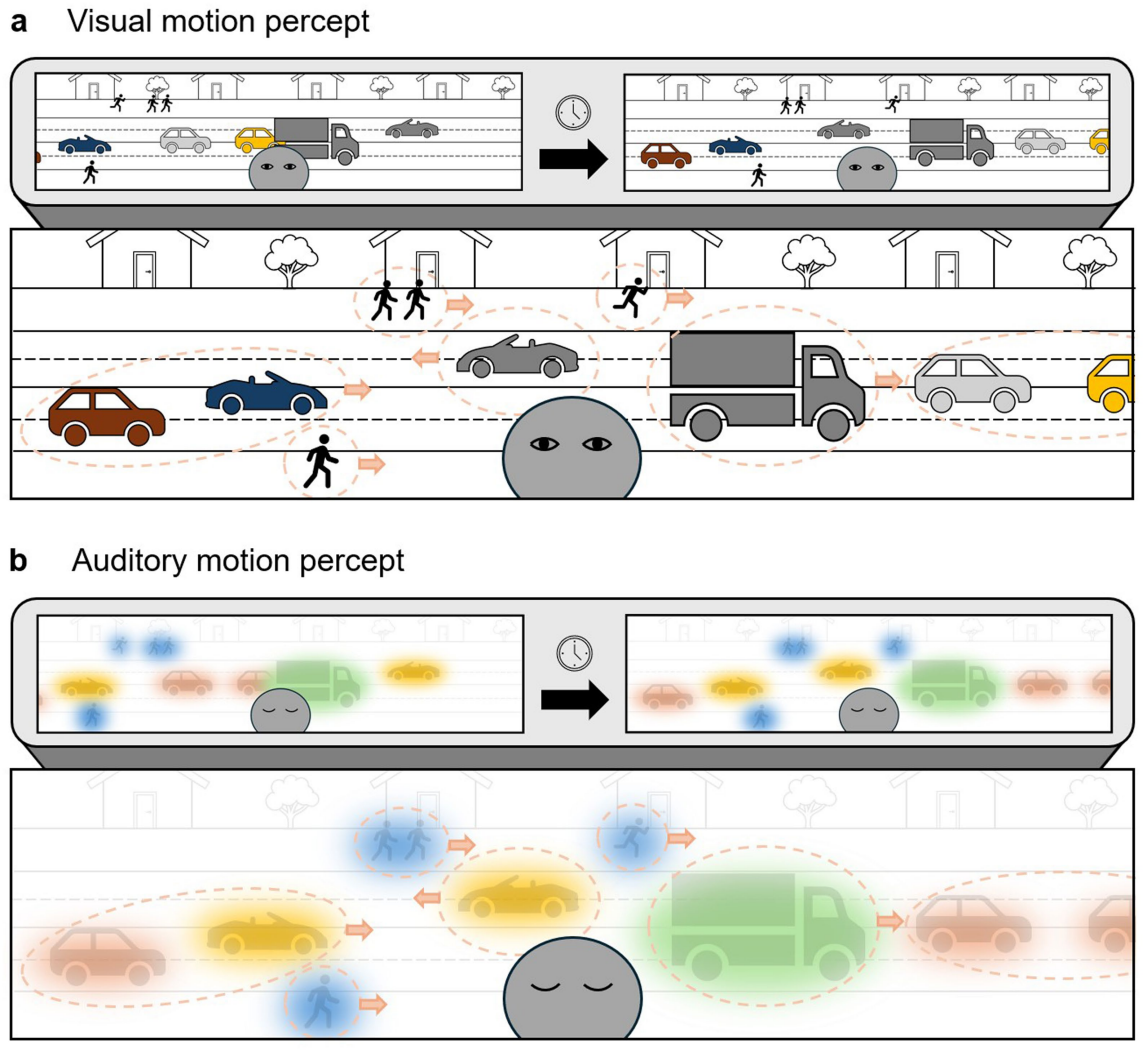
Motion percept in everyday life. Perceiving objects in motion is crucial for interpreting our environment. We establish correspondence between similar objects across space and time, indicating which object is moving in which direction **(a)**. This illustrates how correspondence could be established based on visual information, such as the shape, color, and time-dependent position **(b)**. This illustrates how correspondence could be based on auditory information, such as the object’s sound and time-dependent position.

How exactly the correspondence mechanism works is an ongoing research topic. This question is often investigated using apparent motion displays, in which two stationary objects that appear and disappear one after the other are perceived as one moving across space (Korte, 1915; Wertheimer, 1912). Previous studies showed that one major factor in visual and auditory modalities to perceive good apparent motion is the spatiotemporal distance between objects (e.g., Korte, 1915; Lakatos, 1993; Wertheimer, 1912). In particular, the distance travelled between the observations must be appropriate in relation to the time passed. Therefore, if an object travels too far in too short a time, no correspondence would be established between the object instances, causing us to perceive two different objects instead of one object moving across space, even if they are very similar in other regards. However, establishing correspondence between objects is not always straightforward, for example, when the trajectories of the objects cross. How do we know which object is which after they have intersected? This is particularly difficult if due to occlusions we only have two observations of this scene, i.e., the moment before they intersect and the moment after. How our sensory systems establish correspondences between objects in such ambiguous cases can provide insights into how the underlying correspondence mechanism works. Figure 1 illustrates this mechanism with examples of the resulting visual and auditory apparent motion percept.

**Figure 2:**
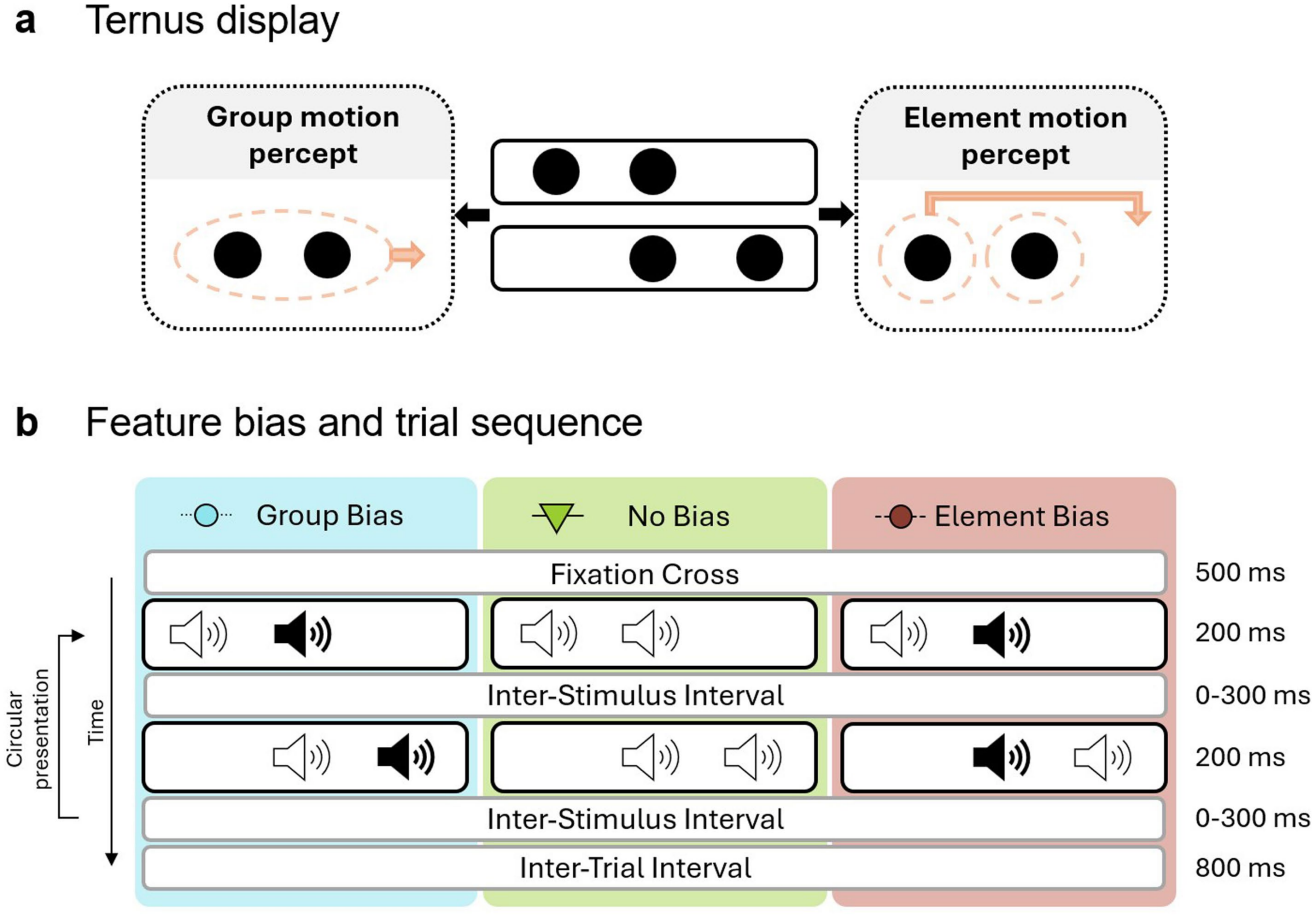
Illustration of the Ternus display. **(a)** Illustration of a visual Ternus display and its two possible motion percepts. In the case of a group motion percept, the stimuli are perceived as a group which moves together to the right. In the case of the element motion percept, the center stimulus is perceived as stationary while the outer stimulus seems to jump over the center one to the other side. **(b)** Illustration of the feature bias in an auditory Ternus display and the trial sequence used in the experiment. The no bias condition (green box) shows an unbiased display, in which all tones had the same feature. In the group bias condition (blue box), the feature of the left tone in each frame differed from the right tone (here illustrated with different colors). In the element bias condition (red box), the feature of the center tones differed from the feature of the left and right tones.

The Ternus display (Ternus, 1926), as an example of an ambiguous apparent motion display, is a helpful tool to investigate correspondence mechanisms across modalities (see Figure 2a). In the visual version of this display, two stimuli appear on the left and at the center of the screen (frame 1), then the stimuli disappear for a variable break (inter-stimulus interval, ISI) and reappear afterwards at the center and on the right (frame 2). This display is ambiguous as the viewer can either perceive that the two stimuli moved to the right together as a group (group motion), or that the left stimulus moved across the center stimulus to the right while the center stimulus remained stationary (element motion). In the case of group motion, the visual system established correspondence between the first stimulus in each frame and between the second stimulus in each frame. In the case of element motion, the visual system established correspondence between the stimuli at the center and between the outer stimuli, resulting in a completely different interpretation of the scene. Both motion percepts are illustrated in Figure 2a. Manipulating the ISI between the stimulus frames shows that spatiotemporal information can influence the apparent motion percept in the Ternus displays (e.g., Pantle and Picciano, 1976; Petersik and Pantle, 1979). In particular, short ISIs lead to more element motion, whereas long ISIs lead to more group motion percepts. In addition, correspondence in the Ternus display also depends strongly on the feature similarities of the stimuli (e.g., Hein and Moore, 2012; Kramer and Yantis, 1997). This has been shown by adding a feature bias to the Ternus display, like for example changing the size/shape, color and orientation of the Ternus elements in a way that is compatible with the element or the group motion percept (e.g., Hein and Moore, 2012; Kramer and Yantis, 1997; Petersik and Rice, 2008). For example, for an element bias the second stimulus in the first frame and the first stimulus in the second frame shared the same feature, which was different from the other two stimuli. For a group bias, the first stimuli of each frame shared the same feature, which was different from the second stimulus. The studies showed that the percept was shifted in the direction of the bias, i.e., in the element bias condition more element motion was perceived and in the group bias condition more group motion was perceived. In addition to these effects of feature information available at relatively low levels of visual processing, feature information available only at higher levels of processing has also been shown to influence the visual Ternus percept. This includes perceived size or lightness and the grouping strength between stimuli (e.g., Alais and Lorenceau, 2002; He and Ooi, 1999; Hein and Moore, 2014; Stepper et al., 2020). Thus, correspondences between visual objects can be based on spatiotemporal information, as well as low-level and higher-level feature information.

Several theories have been developed based on the findings that spatiotemporal and feature information influence correspondence in the visual modality. There are two broad categories: motion-based and object-based theories (Hein, 2017). These two types of correspondence theories focus on different factors and assume different processing levels for the correspondence mechanism. One example of a motion-based theory is the spatiotemporal filter theory, which suggests that low-level spatiotemporal filters determine the direction of motion energy between stimuli, thus determining how apparent motion is perceived (e.g., Adelson and Bergen, 1985; van Santen and Sperling, 1985). Motion-based theories focus on factors available at an early processing level, such as the time delay between stimulus presentations (i.e., ISI in the Ternus display) and the spatial distance between Ternus elements, because those factors influence the spatiotemporal luminance contrast of the stimuli. However, motion-based theories are blind to higher-level feature information and other higher-level factors. This is why object-based theories have been developed. These theories focus on the relationship between objects and their surroundings as well as on perceived feature information. Hein and colleagues (e.g., Hein and Cavanagh, 2012; Hein and Moore, 2014), in particular, assume that correspondence is based on connecting the objects that are perceived as most similar to each other. Together these theories suggest that correspondence in the visual modality occurs at different levels of visual processing and uses the available information at specific levels. Therefore, spatiotemporal information (i.e., the ISI), plays a greater role in motion-based theories, whereas perceived feature information and the relationship between objects play a greater role in object-based theories.

Everyday objects of course do not exist purely visually, as most objects also make sounds. When we process the incoming auditory information, we filter and group sounds, resulting in the perception of auditory streams (auditory scene analysis; e.g., Bregman, 1990). Stream segregation is influenced by feature information, such as the sound’s frequency and timbre (e.g., McAdams and Bregman, 1979; van Noorden, 1975), as well as temporal information, such as the presentation rate at which the tones follow each other (e.g., van Noorden, 1975). In general, sounds with small feature differences and a low presentation rate are perceived as one stream, while sounds with large feature differences and a high presentation rate are perceived as different streams (e.g., Bregman, 1990; McAdams and Bregman, 1979; van Noorden, 1975). However, what factors influence auditory apparent motion perception is not that well researched. Lakatos (1993) demonstrated that apparent motion between the sounds of two loudspeakers can be perceived when the spatial separation between the loudspeakers and the time between the sounds is neither too long nor too short. Thus, simple apparent motion between sounds appears to be based on very similar spatiotemporal constraints as in the visual domain. To understand how correspondence works in the auditory modality, however, it is important to examine the apparent motion percept in ambiguous situations, because the correspondence problem is more apparent in this case. Specifically, rather than distinguishing between motion and no motion as in simple apparent motion, in ambiguous apparent motion the perceptual system is confronted with a real choice between different motion percepts and, thus, different solutions to the correspondence problem. To investigate this in the auditory modality, we can use an auditory version of the Ternus display. Wang et al. (2014) created such a display by replacing the visual stimuli with sounds from three loudspeakers (placed to the left, at the center and to the right in front of the observer). The presentation sequence was similar to the visual Ternus display, i.e., in frame 1 a sound was emitted from the left and center loudspeaker and after the ISI frame 2 followed during which a sound was emitted from the center and right loudspeaker. Wang et al. (2014) manipulated the duration of the ISI and found that the spatiotemporal factor influences the auditory Ternus. Kriegeskorte et al. (2026) recently investigated whether a feature-based bias influences the percept in the auditory Ternus. In particular, they manipulated the frequencies of sinewave tones (500 and 1,000 Hz) creating an element and a group bias (see Figure 2b). They showed that listeners perceived more group motion in a group bias condition compared to an element bias condition. Kriegeskorte et al. (2026) concluded that spatiotemporal as well as feature information can influence ambiguous apparent motion in the auditory Ternus display, similar to in the visual modality.

The study by Kriegeskorte et al. (2026) showed that creating feature biases with different frequencies of pure sinewave tones affected the auditory Ternus percept. This study aims to determine if this finding generalizes to more complex sounds. As complex sounds are more meaningful to us, they might involve higher levels of processing, in a similar way as (perceived) feature information does in the visual modality. Watson and Kidd (2007), for example, showed that the familiarity of the sounds could influence sound discrimination. If complex sounds are processed at a higher level, this could activate an object-based auditory correspondence process that focuses on feature information, similar to the distinction that exists in the visual modality. This process may increase the strength of the feature effect and reduce the effect of the ISI compared to the less complex sinewave tones used by Kriegeskorte et al. (2026). To test this, we used sounds from musical instruments, a guitar sound of an E4 and a piano sound of a C5. These complex sounds combine different frequencies and different timbres, i.e., varying overtones and fundamental tones, instead of just different fundamental tones, as is the case with sinewave tones. Using these two different sounds of musical instruments we created two types of feature biases, an element motion bias and a group motion bias. We also manipulated the spatiotemporal information between the auditory frames. In line with Wang et al. (2014) and Kriegeskorte et al. (2026), we expected to replicate the spatiotemporal influence, i.e., to find more group motion percepts with longer ISI. In addition and most importantly, if complex sounds influence how we establish auditory correspondence, the bias based on the complex feature information should influence correspondence, leading to more group motion percepts in the group bias condition and more element motion percepts in the element bias conditions (e.g., Hein and Moore, 2012; Kramer and Yantis, 1997; Kriegeskorte et al., 2026; Petersik and Rice, 2008). Finding a feature effect for complex sounds would suggest that, as in the visual modality, different types of feature information can be used to solve auditory correspondence. Moreover, if the more complex and meaningful stimuli involve object-based correspondence, the feature effect may increase and the ISI effect may decrease compared to simple sinewave tones.

## 2 Materials and methods

### 2.1 Participants

Twenty-four observers (16 females; aged between 19 and 31; average age 22.38 years; 23 right-handers) participated in the experiment. The sample size was based on power analyses that used the *F*-value, the degrees of freedom and the adjusted partial $\eta^{2}$ (Mordkoff, 2019) from the study by Kriegeskorte et al. (2026, Exp. 2). For the main effect of feature bias, a sample size of 22 was calculated to achieve 80% of power, assuming an alpha of 5%. Therefore, we planned a sample size of 24. All participants reported that their vision and hearing were normal or corrected-to-normal. Participants who indicated perceiving more element motion with longer ISI (i.e., an inversive function of ISI) were replaced (two participants), as is usually done in studies with the visual Ternus display (e.g., Hein and Moore, 2012; Kramer and Yantis, 1997). In addition, participants who perceived no motion in more than 30% of the trials were replaced (one participant). Typically, in each condition it should be possible to perceive motion, thus participants who report that much no motion percepts very likely misunderstood the task. As compensation for the study, participants received either course credit or money (12 Euro/h). All participants agreed to a consent form that complies with the ethical guidelines of the Declaration of Helsinki (World Medical Association, 2013). The Ethics Committee for Psychological Research of the University of Tübingen approved the experiment (reference number: Labor_Rolke_2022_0413_252).

### 2.2 Equipment, stimuli, design, and procedure

Three mini stereo speaker sets (Trust Leto Compact 2.0 Speaker Set; audio input: 3.5 mm; dimensions: 73 × 62 × 55 mm) were arranged on a horizontal line 30 cm in front of the participants, facing upwards. One set was positioned to the left, one at the center, and one to the right, with 45 cm between the sets. Speakers within each set were aligned directly behind each other and concealed behind a cardboard panel to prevent location-based expectations. A computer monitor (70 cm viewing distance; resolution: 1920 × 1,200 resolution; 59.95 Hz refresh rate) was placed behind the speakers. Stimulus presentation was controlled by a Hawlett-Packard HP Compaq 8,200 Elite CMT PC (Intel Core™ i3-2100 CPU, 3.10 GHz, Mesa Intel HD Graphics 2000) running Ubuntu 22.04 LTS. The Creative Sound Blaster Audigy 2 sound card enabled multiple outputs. The experiment was programmed in Matlab (Mathworks Inc., MA, USA, Version R2022a) using Psychophysics Toolbox 3 (Brainard, 1997; Kleiner et al., 2007; Pelli, 1997), with each speaker set controlled independently via PsychPortAudio functions.

We presented 200 ms tones (volume of 70 dB, measured at a distance of 5 cm) at three different loudspeaker locations (left, center, and right) in a horizontal line in front of the participants. To ensure that there was no pause between the middle tones with an ISI of 0 ms, a tone with a length of 400 ms was played in this case, which changed its feature after 200 ms if necessary. To avoid sound mixing within a frame, we used an offset between the first and second tone (80 ms; within-frame interval, WFI, based on Kriegeskorte et al., 2026). To introduce a feature-based bias we presented a guitar and piano sound in a way that the first tone of each frame had another feature than the second tone (group bias), or the outer tones had another feature than the middle tones (element bias). In the no bias condition, all tones had the same feature, either guitar or piano. The order of the features (i.e., first guitar then piano sound or vice versa) and the tone used in the no bias condition (i.e., only guitar sounds or only piano sounds) were counterbalanced within a block. Both sounds were artificially generated using the app GarageBand to reduce noise (Apple Inc, 2025). The piano sound was a C5 (fundamental tone had a frequency of 523 Hz) on a virtual grand piano and the guitar sound was an E4 (fundamental tone had a frequency of 330 Hz) on a virtual acoustic guitar. As we used a guitar and a piano sound, we manipulated the timbre of the sounds in addition to the frequency.

A within-subject design was conducted with two factors: ISI with six levels (0, 25, 50, 100, 150, and 300 ms) and Feature bias with three levels (group bias, element bias, and no bias). The presentation of both factors was fully counterbalanced within each block and presented in a random order. Each factor combination of ISI and Feature bias appeared twice per block, resulting in 36 trials per block. Participants completed 15 blocks in total, including one practice block and 14 experimental blocks, which amounted to 540 trials per participant (30 trials per factor combination). The total duration of the experiment was 45 min.

The experiment started with written instructions and a presentation of a visual and an auditory example of element and group motion (with an ISI of zero and a long ISI, 100 ms in the visual example and 300 ms in the auditory example). Afterwards a practice block with no feedback started, followed by the experimental blocks. Each trial started with a 500-ms fixation cross to center participants’ gaze (Figure 2b). Centering the gaze resulted in a central forward-facing head position, which ensured an equal distance between the outer loudspeakers and the observer. This was followed by the auditory stream of the Ternus display: The first frame, the variable ISI and the second frame. The frames were presented in a continuous loop (Kriegeskorte et al., 2026), such that the second frame was followed by the ISI and then the auditory stream with the first frame started again. This was repeated until a response was registered. Participants indicated their motion percept, by pressing the J key for element motion, the F key for group motion or the B key for no Ternus motion. If a key other than those three keys was pressed, the message that an invalid key was pressed appeared on the screen for 1 s. After an inter-frame interval of 800 ms the next trial started.

### 2.3 Data analysis

The data analysis was conducted in RStudio (Version 2023.06.1 + 524, Posit Software, PBC, 2022). The first block was treated as a practice block and was therefore excluded. In addition, trials with invalid keypresses (0.16% across all feature bias conditions; 0.12% within the group bias condition, 0.17% within the element bias condition and 0.17% within the no bias condition) or excessively long reaction times (0.46% of the remaining data across all feature bias conditions, upper cutoff: 11.59 s; 0.67% within the group bias condition, 0.45% within the element bias condition and 0.27% within the no bias condition) were excluded. Excessively long reaction times were defined as those in which the reaction time of a response was above the mean value of all reaction times plus five standard deviations. These trials were excluded as we assumed that participants were distracted from the task in these cases. We calculated the proportion of group motion for each condition and participant. On average, participants reported across all feature bias conditions only in 1.93% of the remaining trials no motion percepts (1.45% within the group bias condition, 0.54% within the element bias condition and 3.80% within the no bias condition), which is why we excluded this answer option from further data analysis.

We used within-subject analyses of variance (ANOVAs) on percent group motion responses, followed by two-sided pairwise *post hoc t*-tests ( α = 0.05). Greenhouse–Geisser corrections (Greenhouse and Geisser, 1959) were applied whenever the sphericity assumption was violated. *Post hoc p*-values were Bonferroni-adjusted. The Cousineau-Morey correction (Cousineau, 2005; Morey, 2008) was used to adjust within-subject standard deviations and standard errors. Shapiro–Wilk tests showed that the group motion response distributions were normal for the Feature bias factor and the interaction of ISI and Feature bias. To compare the strength of the feature and the ISI effect in our current study with those in Kriegeskorte et al. (2026, Exp. 2), we performed a mixed ANOVA on percent group motion responses from both studies (excluding the no motion percepts in the data by Kriegeskorte et al. (2026) for better comparison with our data). The ANOVA results were followed up by two-sided Welch-*t*-tests for the between-subject comparisons (Bonferroni-adjusted).

## 3 Results

**Figure 3:**
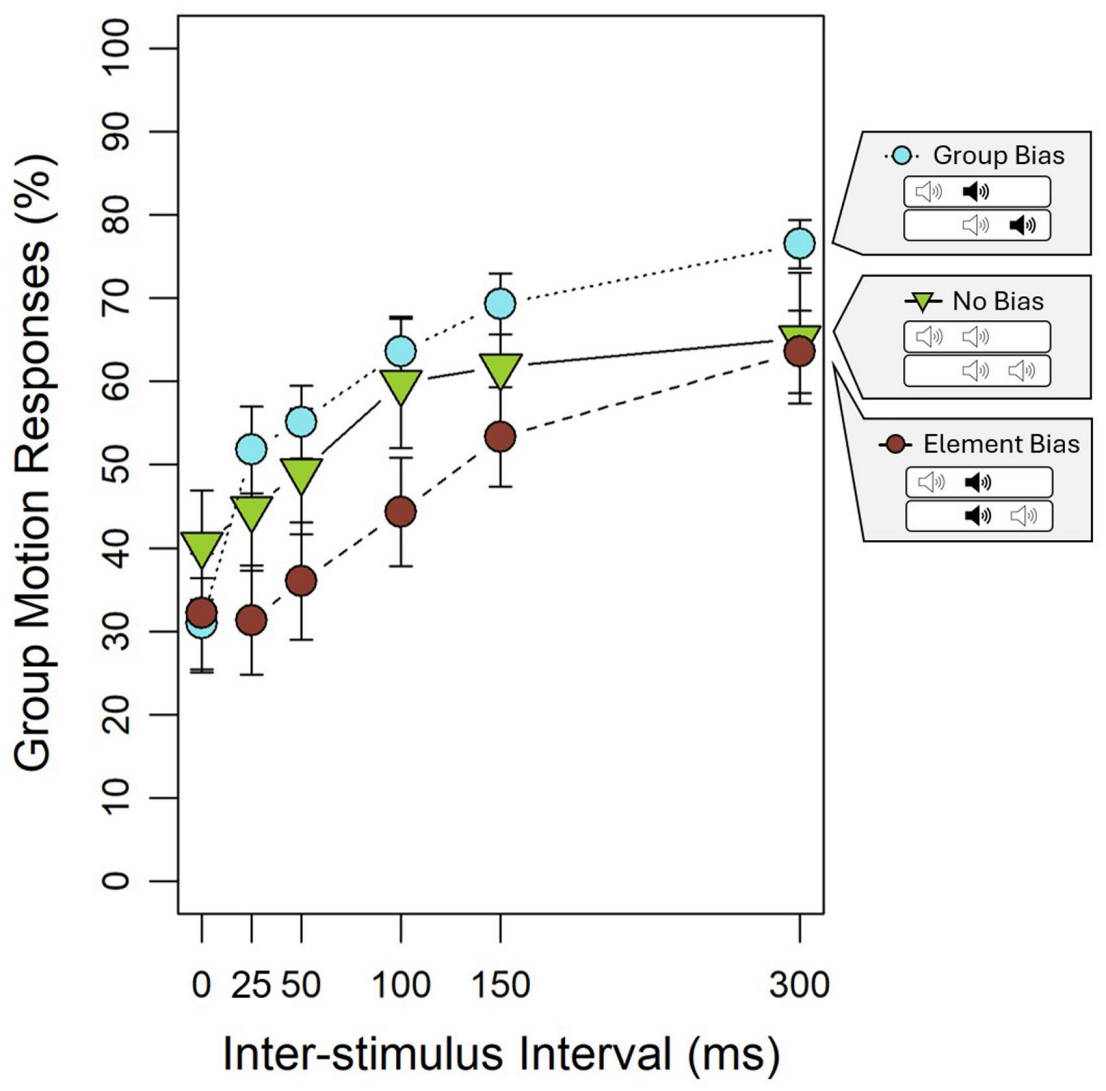
The influence of spatiotemporal information and feature bias on perceived group motion. Mean percent of perceived group motion as a function of ISI and feature bias (group, element, and no bias). The error bars represent within-subject standard errors (SE) by Cousineau-Morey.

In Figure 3, mean group motion responses are illustrated as a function of ISI and Feature bias. A two-factorial 6 (ISI: 0, 25, 50, 100, 150, and 300 ms) × 3 (Feature bias: group bias, element bias, and no bias) repeated-measures ANOVA on individual mean group motion responses showed a significant main effect of ISI, *F*(5, 115) = 33.03, *p* < 0.001, $\eta_{p}^{2}$ = 0.59, as group motion percepts increased with increasing ISI. Post hoc t-tests revealed that all adjacent ISI levels were significantly different from each other, *t*s(23) ≥ 3.40, *p*s ≤ 0.012, *d*s ≥ 0.69. The analysis showed no main effect of Feature bias, *F*(2, 46) = 1.23, *p* = 0.228, but a significant interaction between ISI and Feature bias, *F*(10, 230) = 3.94, *p* = 0.011, $\eta_{p}^{2}$ = 0.15.

To analyze this interaction further, two-factorial repeated-measures ANOVAs were conducted for only two feature bias conditions at a time. For the group and element bias conditions we found a significant feature bias effect, *F*(1, 23) = 6.44, *p* = 0.018, $\eta_{p}^{2}$ = 0.22, showing more group motion responses for the group bias condition compared to the element bias condition, as expected. We also found a significant interaction between the ISI and the Feature bias, *F*(5, 115) = 5.51, *p* = 0.004, $\eta_{p}^{2}$ = 0.19, as the feature bias was significant for medium ISI (25 ms, 50 ms and 100 ms), *t*s(23) ≥ 3.08, *p*s ≤ 0.032, *d*s ≥ 0.63, and decreased with longer ISI, as the trend for the two longest ISI conditions indicated, *t*s(23) ≥ 2.66, *p*s ≤ 0.084, *d*s ≥ 0.54. For the 0 ms ISI condition there was no significant feature bias effect, *t*(23) = 0.13, *p* = 1. Analyzing only the element and the no bias conditions revealed no feature bias effect, *F*(1, 23) = 0.67, *p* = 0.421, but a significant interaction between the ISI and Feature bias, *F*(5, 115) = 3.26, *p* = 0.029, $\eta_{p}^{2}$ = 0.12. *Post hoc* comparisons for each ISI step, however, showed no significant effects, *t*s(23) ≤ 1.19, *p*s = 1. Analyzing only the group and no bias conditions revealed again no main effect of Feature bias, *F*(1, 23) = 0.23, *p* = 0.636, and only a trend for the interaction between the ISI and Feature bias, *F*(5, 115) = 3.12, *p* = 0.058, $\eta_{p}^{2}$ = 0.12. Post hoc comparisons did not reveal significant effects for any ISI level, *t*s(23) ≤ 1.30, *p*s = 1.

For comparing the strength of the feature and the ISI effect, we conducted a three-factorial 6 (ISI) × 3 (Feature bias) × 2 (Sound type: simple and complex sounds, between experiments factor) mixed ANOVA on individual mean group motion responses. The ANOVA revealed no significant main effect of the Sound type, *F*(1, 40) = 0.39, *p* = 0.535, and no significant interaction between the Sound type and the Feature bias, *F*(2, 80) = 0.38, *p* = 0.604, indicating that the strength of the feature bias did not differ between the experiments. However, there was a significant interaction between the Sound type and the ISI, *F*(5, 200) = 9.51, *p* < 0.001, $\eta_{p}^{2}$ = 0.19, as the ISI had a stronger influence on the motion percept with the simple sounds than with the complex sounds. Post hoc Welch-*t*-tests confirmed a significant difference between the two sound types at the longest ISI, *t*(36.12) = 3.72, *p* = 0.004, *d* = 1.17, in which the percentage of group motion responses was higher for the simple than for the complex sounds. At any other ISI, the sound types did not differ, *t*s ≤ 2.35, *p*s ≥ 0.144. Finally, the ANOVA revealed a trend for a three-factorial interaction between Sound type, Feature bias and ISI, *F*(10, 400) = 1.96, *p* = 0.090, $\eta_{p}^{2}$ = 0.05. This is in line with the feature bias being descriptively shifted towards longer ISIs with complex sounds compared to simple sounds.

## 4 Discussion

Auditory correspondence allows us to perceive auditory information emanating from an object as moving from one position to another because the auditory information can be used to connect the right objects across space and time. We used an auditory version of the Ternus display (Kriegeskorte et al., 2026; Wang et al., 2014) and manipulated the ISI. In addition, we introduced a feature bias using piano and guitar sounds that were either compatible with the element motion or the group motion percept. Our goal was to replicate and generalize the feature-based bias identified by Kriegeskorte et al. (2026) with simple sinewave tones to more complex sounds. Furthermore, we examined whether incorporating complex, meaningful features alongside lower-level sound characteristics increases the feature effect and reduces the ISI’s impact on the correspondence solution, as an object-based correspondence mechanism would suggest. Our results demonstrate that auditory correspondence is influenced by both spatiotemporal information as well as the features of the auditory stimuli. These results replicate those of Kriegeskorte et al. (2026) and demonstrate generalizability by showing that different types of feature information influence auditory correspondence.

A closer look at the comparison between this study and Experiment 2 from Kriegeskorte et al. (2026) also revealed some interesting differences in the exact pattern of results. First, the feature bias effect for complex sounds in the current experiment was descriptively largest at long ISIs and decreased for shorter ISIs, while in the study by Kriegeskorte et al. (2026) the reversed pattern was present for sinewave tones (the three-way interaction, however, only showed a trend). The temporal shift of the feature bias effect toward longer ISIs for complex sounds may be due to participants needing more time to perceive and identify complex sounds. Second and more importantly, the influence of the ISI was weaker for complex musical sounds than for simple sinewaves. This result is compatible with the assumption that processing complex sound features relies on higher-level, object-based processing, in which the impact of low-level factors, such as the ISI, is reduced. However, in line with this interpretation, we would have expected the feature bias to be stronger for complex sounds than for simple sounds, which was not the case. Therefore, the evidence for the existence of an object-based correspondence mechanism in the auditory modality is mixed. Future studies should investigate the influence of higher-level feature information in more detail. For example, they could use stimuli that are identical at the physical level but differ at a higher level of processing or they could manipulate the cognitive demands of the task.

In summary, our results showed that spatiotemporal and feature information play an important role in connecting auditory objects across space and time. The findings replicated and generalized the results of Kriegeskorte et al. (2026) to another type of feature information. Furthermore, our results suggest that higher-level processes might be involved for solving auditory correspondence.

## Data Availability

The raw data supporting the conclusions of this article are available at zenodo.org (https://doi.org/10.5281/zenodo.20139845).
